# Should adults with diabetes mellitus be vaccinated against hepatitis B virus? A systematic review of diabetes mellitus and the progression of hepatitis B disease

**DOI:** 10.1080/21645515.2017.1353850

**Published:** 2017-07-25

**Authors:** Zobair Younossi, Katrin Kochems, Marc de Ridder, Desmond Curran, Eveline M. Bunge, Laurence de Moerlooze

**Affiliations:** a Center for Liver Disease, Department of Medicine, Inova Fairfax Hospital, Falls Church, VA, USA; b Pallas Health Research and Consultancy, Rotterdam, The Netherlands; c Faculté de Pharmacie, Université Libre de Bruxelles, Bruxelles, Belgium; d GSK, Wavre, Belgium

**Keywords:** cirrhosis, diabetes mellitus, hepatitis B virus, hepatocellular carcinoma, hepatitis B, liver disease, NASH, systematic, Type 2 diabetes mellitus

## Abstract

Despite the burden of diabetes mellitus (DM), little is known about the role of this and other metabolic syndromes on the severity of hepatitis B virus (HBV) chronicity and liver disease progression. The value of hepatitis B vaccination and its impact on liver diseases and HCC has been largely demonstrated, adult vaccination coverage is however suboptimal and DM diagnosis represents an opportunity for the HCP to discuss hepatitis B and other adult vaccinations.

We performed a systematic literature search to identify studies (January 2000 to January 2017) describing liver disease progression among patients with HBV by DM status. Risk factors were assessed including the relationship between HBV and non-alcoholic steatohepatitis (NASH). Data were extracted systematically and assessed descriptively.

Twenty articles described liver disease progression and one article evaluated NASH among subjects with HBV by DM status. Fourteen articles reported that DM as a predictor for the outcome, including delayed seroclearance, cirrhosis, hepatocellular carcinoma, transplant/mortality and death, whereas no association on liver outcomes was found in 7 studies.

In summary, our review suggests that DM is associated with the progression of severe liver outcomes in adults with HBV, although more studies are needed to understand the benefits of HBV vaccination in adults with DM and liver-diseases.

## Introduction

Infection with hepatitis B virus (HBV) can cause acute and chronic hepatitis, and complications such as cirrhosis and hepatocellular carcinoma (HCC).^1,2^ In HBV-endemic regions such as East Asia and sub-Saharan Africa, up to 10% of the adult population have chronic HBV infection, whereas the prevalence in Western Europe and North America is <1%.^1,2^ Vaccines against HBV have been shown to provide protection, and in HBV-endemic countries, universal vaccination against HBV has substantially reduced HBV-related liver disease and mortality.^3^ In countries where the prevalence of HBV infection is relatively low, the inclusion of HBV on childhood immunization programs has had a measurable impact on HBV-related liver disease.^3^ However, HBV vaccination is usually implemented on a risk-based approach in adults, and there remains debate about which adult groups to target to optimize the clinical benefits and cost-effectiveness of vaccination in non-endemic countries.

People with diabetes mellitus (DM) require blood glucose monitoring and are at risk of blood-borne pathogens such as HBV. Although there are reports that lapses in infection control have been associated with HBV transmission in healthcare and institutional settings, studies in the US also show that HBV rates are higher in adults with DM compared with adults without DM in the general population.^4^ In a National Health and Nutrition Examination Survey, the prevalence of HBV infection in people with DM was 60% higher than in those without DM,^4,5^ and in an Emerging Infections Program study in the US including 64.2 million people from 2009 to 2010, adults with DM had about twice the risk for acute HBV infection than adults without DM.^4,5^

Although DM is an established risk factor for numerous co-morbidities including chronic liver disease, a role for HBV-related liver dysfunction in the development of Type 2 DM has recently emerged. It has been proposed that viral hepatitis may impair key metabolic processes regulated by the liver which are implicated in the development DM based on the interplay of inflammatory mechanisms induced by infection of the liver, ultimately leading to glycometabolic dysfunction and insulin resistance.^6-11^ In a recent meta-analysis, people with HBV infection versus without HBV were at a higher risk of developing DM.^4^

Adults with DM have an increased risk of HCC and cirrhosis, and other hepatic disorders such as non-alcoholic fatty liver disease (NAFLD), including the progressive form, non-alcoholic steatohepatitis (NASH).^12-15^ NAFLD is being increasingly recognized as the liver disease component of metabolic syndrome, and NASH can potentially lead to liver fibrosis, cirrhosis, failure, and HCC.^16-18^ Moreover, fatty livers are vulnerable to injury by viruses and hepatotoxic injury, and patients with hepatic steatosis often present with hepatitis C virus (HCV) infection; as well as metabolic syndrome in the host, there is evidence that the progression of fatty liver disease has a viral etiology associated with the HCV genotype.^19-21^ The association between fatty liver disease and HBV is less clear, with various studies reporting that fatty liver disease in people with HBV is largely a result of components of metabolic syndrome in the host.^22-24^ However, in a large population-based study in Taiwan, the correlation between HBV and elevated liver biochemical parameters in patients with fatty liver disease was not associated with age and obesity, suggesting a viral etiology.^23^

In the US, HBV vaccination is recommended for adults with DM aged 19–59 years,^25^ and in Belgium, HBV vaccination is recommended for adults with DM aged 23–59 y.^26^ However, in most countries with a relatively low prevalence of HBV infection, HBV vaccination in adults is reimbursed on a risk-based approach with no specific guidance on DM.

Despite the high global burden of DM, little is known about the interplay between HBV and metabolic syndromes, and how this influences the progression to severe liver disease. To our knowledge, the association between DM and HBV disease has not been reviewed using an evidence-based systematic approach to date. We undertook this systematic review to analyze the strength of the evidence on this topic with the following objectives: (1) to describe disease progression of subjects with HBV infection by DM status and associated risk factors (seroconversion, seroclearance, cirrhosis development, decompensated cirrhosis, HCC, liver transplant, and death) and (2) to understand the relationship between HBV, NAFLD, and NASH. The aim of analyzing these outcomes was to describe metabolic syndromes and HBV-related disease to help understand the value of HBV vaccination in adults with DM

## Results

### Literature search

**Figure 1.**
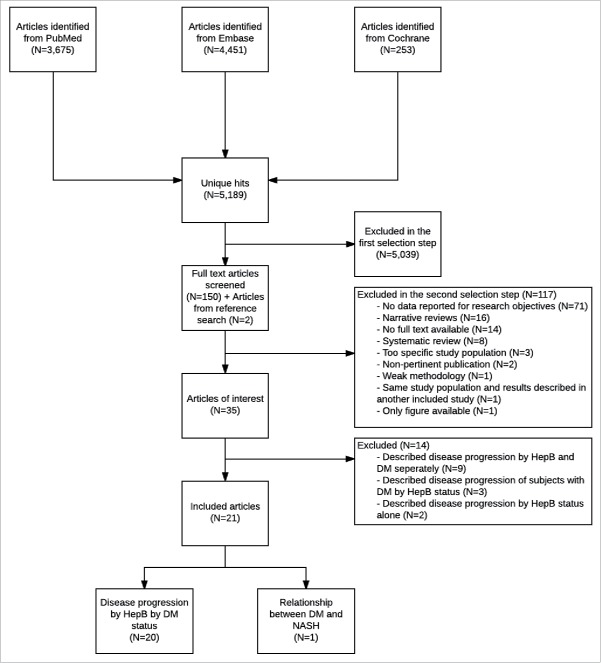
Selection of articles DM: diabetes mellitus; HepB: hepatitis B; NASH: non-alcoholic steatohepatitis.

A total of 5,189 unique articles were identified from the initial literature search but no data from the gray literature. After screening titles and abstracts, the full text from 152 articles were examined in detail which identified 35 articles of interest (Fig. 1). Common reasons for exclusion at this step included absence of data on research objectives (n = 71), narrative reviews (n = 16), unavailability of full text (n = 14), and systematic reviews (n = 8). At the start of the selection process, articles describing only data for a DM population or only for an otherwise healthy population were selected for full-text screening, but were excluded from data extraction, because sufficient articles comparing disease progression by DM status were identified. As these direct comparisons provided the most robust data, 14 non-comparative articles were excluded, providing a total of 21 articles for inclusion in the review.

Twenty articles described the disease progression of subjects with HBV by DM status and one article dealt with the prevalence of NASH within subjects with HBV. The studies were conducted in the following countries: Taiwan (n = 8), China (n = 5), US (n = 3), Korea (n = 1), Spain (n = 1), Greece (n = 1), New Zealand (n = 1), France (n = 1) and Spain (n = 1). The majority of the articles presented data for only one outcome and adjustments were made for one or more potential confounding factors in all studies. The study designs were cohort (n = 16); case-control (n = 3), nested case-control (n = 1), and cross sectional (n = 1). Eleven articles described liver disease progression in Type 2 DM patients, while DM type was not described in the remaining articles. The study characteristics are shown in Table 1.

**Table 1.** Design of studies.

Country		Design	
Study period		• Inclusion criteria	
Reference	Study population	• Exclusion criteria	Limitations
China	Ethnic Chinese CHB patients with liver biopsy or transient elastography between 2005 and 2012	Cohort	Kaplan–Meier and Cox regression analysis based on age at HBeAg seroclearance as the end point instead of time from baseline to HBeAg seroclearance; effect of metabolic risks modification during the follow-up period on immune virological response and eventually clinical outcomes not determined
2005–2013		• HBsAg positive for > 6 months	
Hsiang et al. 2014^13^		• HCV or HIV co-infection; other causes of hepatitis, including autoimmune liver disease, Wilson's disease and haemochromatosis; > 20 g alcohol consumption/day; previous interferon therapy; follow-up period < 12 months	
Taiwan	Adults > 20 y of age with CHB from randomly sampled enrolees from the NHIRD claims data	Cohort	Possibility of misclassification of both the risk and the outcome of interest (diabetes and cirrhosis); unknown validity of the ICD-9 codes to define cirrhosis, esophageal varices, or decompensated cirrhosis; possibility of undetected cirrhosis leading to the development of diabetes; potentially important variables related to diabetes (severity and treatment of diabetes, triglyceride level, and BMI) not included.
1997–2009		• NR	
Huang et al. 2013^14^		• Diagnosis of cirrhosis or esophageal varices before the inception point for follow-up; alcoholic cirrhosis or biliary cirrhosis; alcoholic liver disease or hepatitis C infection	
Taiwan	Patients with a chart-documented history of CHB infection	Cohort	NR
NRHuo et al. 2000^27^		• Seropositive for HBsAg ≥ 6 months; absence of liver cirrhosis; available data for serum biochemical studies and viral serology; at least 2 clinical visits/year; a follow-up for at least one year; non-alcoholism; no previous therapy with interferon or other anti-viral agent	
		• NR	
Greece	HBeAg-negative CHB patients admitted to undergo liver biopsy	Cohort	Causative effect of DM on the histological progression of chronic viral hepatitis not directly supported by data
1998–2003		• NR	
Papatheodoridis et al, 2006^28^		• Malignancy; antiviral or immunosuppressive therapy within the last 6 months; inadequate biopsy; HBV and HCV co-infection; detectable antibodies against hepatitis delta virus or HIV	
China	Patients with CHB identified in primary care clinics and hospitals in different regions of Hong Kong	Cohort	Transient elastography instead of liver biopsy was used as the diagnostic tool to define liver fibrosis. Given that the natural history of liver fibrosis progression occurs over decades, a follow-up period of close to 4 y may still be too short.
2006–2008		• NR	
Wong et al. 2014^29^		• evidence of hepatitis C virus; men who consumed >30 g of alcohol/w and women who consumed >20 grams of alcohol/w; secondary causes of hepatic steatosis; decompensated liver disease; complications of liver surgery/liver transplantation	
Taiwan	> 40 y of age participants from the cancer screening program conducted by the Tainan	Cohort	Use of abdominal girth instead of BMI; patients with fasting blood sugar >126 mg/dL defined as DM cases; pathology reports unavailable; no active surveillance
2004–2007	County Health Bureau	• NR	
Chen et al. 2013^31^		• Participants with incomplete data or HCC at baseline were excluded	
Taiwan	Residents ≥ 35 y old	Cohort	Abdominal ultrasonography screening not performed for seronegative participants;
1997–2004		• NR	Possibility of overestimating the risk of developing HCC in viral hepatitis–positive participants; effect of antiviral therapies on development of HCC not investigated; small sample size for incident HCC cases
Wang et al. 2009^34^		• Diagnosed with HCC before the screening	
Taiwan	Participants of cancer screening program held between 1991 and 1992	Cohort	Self-report of diabetes status; no information on high-density lipoprotein cholesterol levels, and blood pressures; small numbers of HCC cases with extreme obesity; risk factors based on BMI measurement at enrolment; definition of extreme obesity (BMI 30 kg/m2) may not apply globally
Chen et al. 2008^12^		• NR	
		• Subjects with liver cancer before/at enrollment	
China	Cases: Hospitalized patients with HBV related HCC at Jinan Infectious	Case-control	Overweight and obese subjects not included; all HCC and cirrhosis subjects not diagnosed histologically.
2004–2008	Disease Hospital	• Hospitalized for HCC or CHB; ≥ 30 y of age; HBsAg positive; anti-HCV negative; no history of cancer other than HCC or hepatitis other than hepatitis B; no cancer treatment; no treatment with nucleotide/nucleosides or interferon; residence of Shandong Province	
Li et al. 2012^15^	Controls: CHB patients without HCC hospitalized at Jinan Infectious	• NR	
	Disease Hospital (hospital cross-sectional CHB controls)		
Taiwan	Cases: All hospitalized inpatients first diagnosed with HCC in the computerized database	Case-control	Biased control group in terms of risk factors in association with HCC; difficult to investigate the relationship between DM and HCC; no information on fasting insulin or postprandial glucose level; effect of antiviral treatment of both HBV and HCV infection not considered; obesity regarded as an independent factor in association with HCC.
2004–2005	Controls: Subjects randomly selected from those participating in the health check-up program at the hospital (controls)	• Cases: ICD-9-CM code 155.0. Controls: NR	
Ko et al. 2012^33^		• Cases: Other primary liver cancer indicated in ICD-9-CM code 155.0 were excluded after reviewing the medical chart; colorectal cancer (ICD-9-CM code 153.0–153.9, 154.0, 154.1 and 154.8), gastric cancer (ICD-9-CM code 151.0–151.9), pancreatic cancer (ICD-9-CM code 157.0–157.8) breast cancer (ICD-9-CM code 174.0–174.9) or lung cancer (ICD-9-CM code 162.0–162.9)	
Taiwan	Enrollees from the NHIRD between 1997–2009	Cohort	Possibility of misclassification of DM and HCC cases; DM diagnosis was not systematically screened at regular intervals; possible under-recognized or under-reported cases; unknown validity of the 9th ICD codes; all variables related to DM or CHB (severity of diabetes, glucose level, HbA1c level, triglyceride level, body mass index, HBV DNA level, HBeAg and family history of HCC) not included; synergism between obesity and alcohol in relation to incident diabetes unknown
1997–2009		• NR	
Fu et al. 2015^32^		• Patients with the HCC diagnosis before the inception point; patients with the diagnosis of cirrhosis, either alcoholic/biliary origin; patients with alcoholic liver disease/hepatitis C infection	
China	Patients diagnosed with LC from June 2003 to July 2013	Cohort	Retrospective design.
2003–2013		• NR	
Xiong et al. 2015^35^		• NR	
Korea	Multicenter, retrospective study of CHB patients who underwent LB and TE before starting antiviral therapy	Cohort	Retrospective design; the number of patients who developed HCC was small (8.9%), which was related to the characteristics of the study population who were receiving antiviral therapy
2005–2015		• NR	
Seo et al. 2016^36^		• Failure to obtain reliable LS values, An invalid LS value; Delay between LB and TE>1 month; Starting antiviral therapy >1 month after LB; Presence/history of HCC at enrolment, HCC development within 6 month after enrolment; History of previous antiviral therapy; History of decompensated cirrhosis, Child Pugh class B/C cirrhosis at enrolment; Unsuitable quality of LB specimen for appropriate interpretation; Coinfection with hepatitis C/D or HIV; Right-sided heart failure; pregnancy	
USA	Non-institutionalized civilian US population with CHB	Cohort	Possibility of liver disease subjects being classified as controls; possibility of underlying NAFLD or ALD in patients with normal liver enzymes; miss-classification of NAFLD cases as ALD due to relatively low threshold for definition of excessive alcohol consumption; histological data absent
1988–2006		• ≥ 17 y of age; complete demographic, social history, and clinical data available	
Stepanova et al, 2010^40^		• NR	
New Zealand	HBV patients with cirrhosis, who sought care at the hospital	Cohort	Retrospective design; possible observational biases and record errors; small size of the cohort; protective effect of metformin on hepatocarcinogenesis not assessed; anthropometric measurement not consistently performed in all patients; assessment for metabolic syndrome un-available
2000–2010		• NR	
Hsiang et al. 2015^37^		• Co-infection with hepatotrophic viruses or HIV; cirrhosis complication in the year before DM diagnosis, type 1 DM patients	
USA	Individuals who were KPNC health plan members in March 1996 and who were diagnosed with hepatitis B either before or after that date	Cohort	NR
1996–2005		• KNPC member between March 1995 and February 1996	
Szpakowski et al. 2013^41^		• Co-infection with HIV or HCV	
Taiwan	Patients who underwent primary liver resection for HCC identified from the Cancer Registry Database of the hospital	Cohort	Small patient number in the dual viral infection
1996–1999		• Complete medical history, including serological markers for hepatitis B and C.; ≥ 6 months follow-up period	
Huo et al. 2003^38^		• NR	
Spain	Cohort: HBsAg positive subjects identified among all blood donors between 1972 and 1985	Nested case-control	Liver morbidity and risk factor exposures not determined in all subjects as 55% of alive HBsAg positive subjects did not participate in the study; HCV and HIV not detected in subjects who died before 1985
1972–2000	Nested case-control study: All HBsAg positive men of the cohort who died because of liver disease (cases) and HBsAg positive men without liver disease (controls).	• NR	
Ribes et al. 2006^39^		• HBV seromarkers negative subjects	
China	Cases: DM patients ≥ 18 y of age who underwent cadaveric related liver transplantation for HBV-related liver disease	Case-control	NR
2003–2007	Control: Liver transplant recipients without DM matched for age, gender, primary liver disease and model for end-stage liver	• NR	
Ling et al. 2011^42^		• NR	
USA	HBsAg positive patients	Cross-sectional	Retrospective design, lack of complete histologic data and HBV genotyping for CHB patients.
2000–2006		• ≥ 18 y of age; negative for anti-HCV and anti-HIV antibodies	
Bondini et al. 2007^43^		• Alcohol intake > 20 g/day; drug use known to cause steatosis such as amiodarone, tamoxifen, methotrexate, tetracycline or corticosteroids	
France, 2008–2013	Patients with chronic hepatitis B infection in in Metropolitan France from January 2008 to December 2013 (largest Western cohort study on the prognosis of patients with CHB to date)	Nationwide, observational cohort study:	No information recorded about CHB treatment
Mallet et al. 2017^30^		• Adults discharged from hospital with a primary or associated diagnosis of CHB (ICD-10 B18.0 or B18.1 codes)	
		• Recipients of solid organ transplant or allogeneic stem cell transplant before January 2008 were excluded	

ALD: alcohol-related liver disease; BMI: body mass index; CHB: chronic hepatitis B; DM: diabetes mellitus; DNA; deoxyribonucleic acid; HbA1c: glycosylated hemoglobin; HBeAg: hepatitis B e antigen; HBsAg: hepatitis B surface antigen; HBV: hepatitis B virus; HCC: hepatocellular carcinoma; HCV: hepatitis C virus; HepB: hepatitis B; HIV: human immunodeficiency virus; ICD: International Classification of Diseases; KPNC: Kaiser Permanente Northern California; LC: liver cirrhosis; LB: liver biopsy; LS: Liver stiffness; TE: Transient elastography; NAFLD: Non-alcoholic fatty liver disease; NHIRD: National Health Insurance Research Database; NR: not reported; US: United States

### Outcomes

#### Seroconversion and seroclearance

None of the articles included in the review presented seroconversion data. The effect of DM on Hepatitis B e-antigen (HBeAg) seroclearance in chronic HBV patients was described in a single article.^13^ This cohort study included 413 Chinese chronic HBV patients who had undergone liver biopsy or transient elastography between 2005 and 2012. After adjusting for viral load, anti-viral therapy and necro-inflammation, DM at baseline was observed to be a predictor of delayed HBeAg seroclearance (Hazard ratio [HR]: 0.55; 95% Confidence Interval [CI]: 0.32, 0.97).

#### Cirrhosis and decompensated cirrhosis

**Figure 2.**
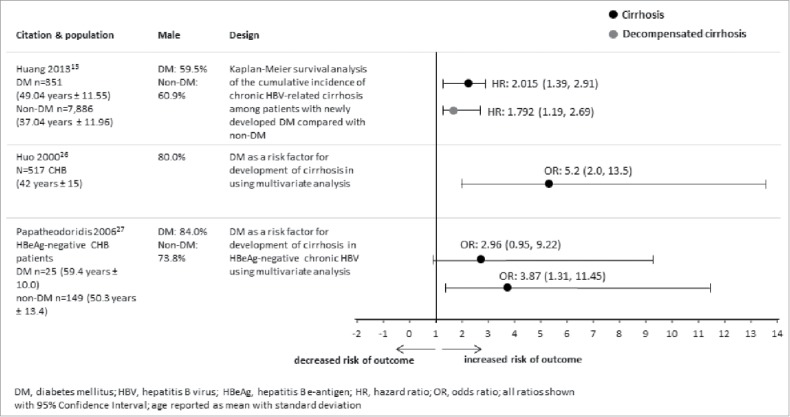
Overview of studies that evaluated DM and HBV-related cirrhosis and decompensated cirrhosis.

An overview of the risk of cirrhosis and decompensated cirrhosis is shown in Fig. 2. Three cohort studies presented the risk of cirrhosis in hepatitis B patients by DM status.^14,27,28^ All 3 studies established that patients with chronic HBV and DM had an increased risk of liver cirrhosis, particularly among the male population. A large population-based cohort study of chronic HBV patients conducted between 1997 and 2009 in Taiwan observed that newly diagnosed DM was an independent predictor for cirrhosis after adjustment for age, sex, HBV treatment, HCC, and co-morbidity index by Cox proportional hazards model (HR: 2.01; 95% CI: 1.39, 2.91).^14^ In another Taiwanese cohort study involving 516 patients with chronic HBV, DM was identified as an independent risk factor of cirrhosis in a multivariate analysis adjusted for age, gender, and persistent hepatitis (odds ratio [OR]: 5.2; 95% CI: 2.0, 13.5).^27^ The third study conducted in Greece between 1998 and 2003, with a cohort of 174 subjects showed that DM was associated with more severe fibrosis in patients with HBeAg negative chronic HBV (model 1 OR: 2.96; 95% CI: 0.95, 9.22; model 2 OR: 3.87; 95% CI: 1.31, 11.45).^28^

A large population-based cohort study of Taiwanese patients with chronic HBV observed that after adjusting for age, sex, HBV treatment, HCC, and comorbidity index by Cox proportional hazards model, DM was an independent predictor for decompensated cirrhosis (HR:1.8; 95% CI: 1.19, 2.7).^14^

One study assessed the impact of DM on liver fibrosis progression in 663 patients with chronic hepatitis B in China.^29^ In patients with type II DM at baseline, after adjusting for viral load, the OR for liver fibrosis progression in those with DM at 44 months follow-up vs. patients with resolved DM was 1.1 (95% CI: 0.5, 2.2: p = 0.87). There was no conclusion regarding liver fibrosis progression by DM status as the main objective of the study was on metabolic syndrome.^29^

A cohort study in France that included 48,189 patients discharged from hospital with a diagnosis of chronic HBV between January 2008 and December 2013, assessed risk factors for liver disease progression, which was a composite outcome of end-stage liver disease and/or hepatocellular carcinoma.^30^ The multivariate analysis showed that DM was a risk factor for liver disease progression with an adjusted HR of 1.40 (95% CI: 1.32, 1.48).^30^

#### Hepatocellular carcinoma

**Figure 3.**
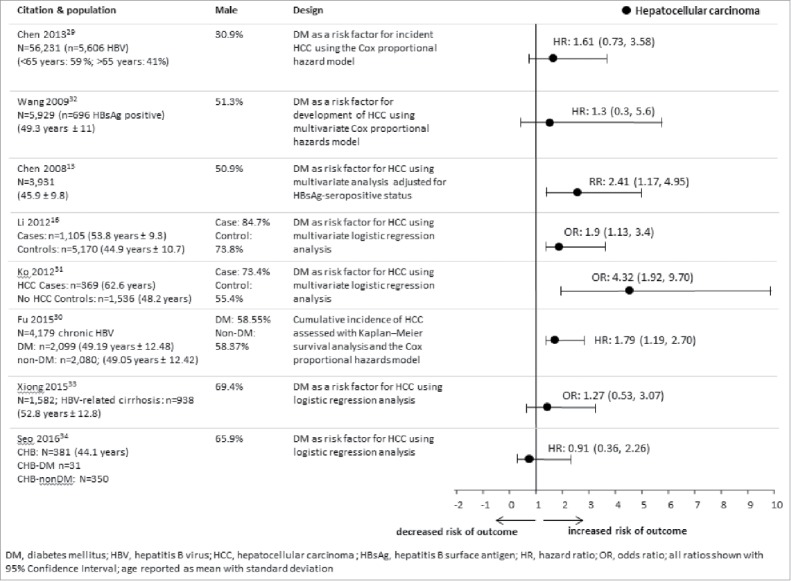
Overview of studies that evaluated DM and HBV-related hepatocellular carcinoma.

An overview of the risk of HCC is shown in Fig. 3. The risk of HCC in hepatitis B patients by DM status was described in 9 studies conducted between 1985 and 2013 in Taiwan (n = 5),^12,31-35^ China (n = 2),^15,35^ Korea (n = 1),^36^ and New Zealand (n = 1).^37^ Six studies used cohort designs, which included the general population,^31,32,34^ chronic HBV patients,^12^ and individuals with hepatitis B-related cirrhosis.^35,37^ One of the studies found that history of DM was the most important risk factor for HCC among hepatitis B surface antigen (HBsAg)-positive subjects.^12^ Another study showed that DM was a significant predictor of HCC in patients with HBV-related cirrhosis.^37^ However, DM was not found to be a significant predictor for HCC in 2 other cohort studies (HR: 1.61; 95% CI: 0.73, 3.58; HR: 1.3; 95% CI: 0.3, 5.6),^31,34^ of which one included only 57 HCC cases which may partly explain the lack of a significant effect.^31^ A further cohort study of predictors of HCC in patients with chronic HBV showed that the proportion of patients with HCC was higher in those with DM than those without (20.6% vs. 6.9%, respectively), and univariate analysis showed that DM was a significant predictor for HCC (p = 0.015).^36^

A cohort study in Taiwan using the National Health Insurance Research Database which included 14,523 chronic HBV patients, found that 3.29% patients in the DM cohort (n = 2099) developed HCC compared with 2.02% in the matched non-DM cohort (n = 2080).^32^ After adjustment for competing mortality, compared with non-DM patients, chronic HBV patients with new onset DM had a significantly higher cumulative incidence of HCC (relative risk: 1.628, 95% CI: 1.114, 2.378).^32^

A retrospective study from China, reviewed the medical records of 1,582 patients diagnosed with liver cirrhosis from June 2003 to July 2013, and found that HCC rates in patients with HBV-induced cirrhosis were higher in DM than non-DM patients (7.1% vs. 6.7%).^35^

Two studies used a case-control design to assess the effect of DM on HCC in patients with HBV.^15,33^ The first study, which included hospitalized HCC cases and a random control sample, observed that the effect of DM on the risk for developing HCC was synergistic with HBV infection.^33^ The second study, which enrolled chronic HBV-related HCC cases and chronic HBV controls, showed that DM was significantly associated with HCC in women with chronic HBV (OR: 1.9; 95% CI: 1.1, 3.4), whereas no association was observed in men.^15^

#### Death and liver transplant

**Figure 4.**
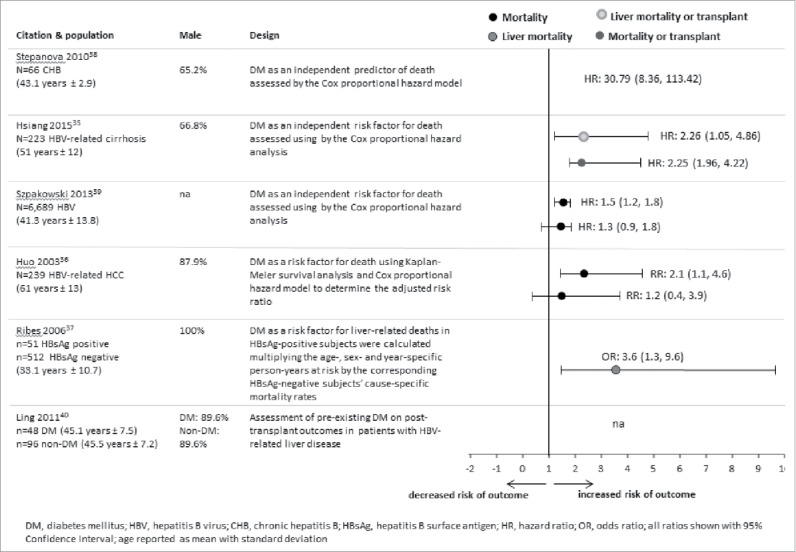
Overview of studies that evaluated DM and HBV-related mortality.

An overview of the risk of death and liver transplant is shown in Fig. 4. Four studies conducted in the US, Taiwan and Spain reported on the risk of death in HBV patients by DM status.^38-41^ Two cohort studies reported all-cause mortality,^38,40^ one nested case-control study reported liver related mortality,^39^ and one cohort study reported all-cause-mortality and HBV-related mortality.^41^ The risk of death in HBV patients was investigated by DM status in 2 of the studies,^38,40^ while in the other 2 studies DM type was not reported.^39,40^ DM was found to be a predictor of all-cause mortality in HBV patients,^38-40^ and DM increased the risk of death from liver disease in HBsAg-positive men.^39^ The cohort study on Kaiser Permanente Northern California members indicated that DM was not a predictor of hepatitis-related death in HBV patients (HR: 1.3; 95% CI: 0.9, 1.8).^41^

Survival rates in HBV patients by Type 1 or 2 DM status were described in 2 studies.^38,42^ A cohort study involving patients who had undergone primary liver resection for HCC in Taiwan during 1996–1999 analyzed survival in HBV-related HCC patients (n = 162).^38^ The survival rate was higher after one, 3, and 5 y in patients without DM compared with those with DM. In a case-control study from China during 2003–2007, the post-liver transplantation survival rate was higher in HBV-related liver disease patients without DM than with DM.^42^ The study also conducted one-, 2-, and 3-year survival rate analyses on DM subgroups, where adult patients with cadaveric-related liver transplant for hepatitis-related liver disease served as cases and liver transplant recipients as controls.^42^

The risk of orthotopic liver transplant combined with the risk of death in HBV patients by DM status was reported from a single cohort study conducted in New Zealand from 2000–2012.^37^ DM was found to be a predictor of all-cause mortality/orthotopic liver transplant (HR: 2.25; 95% CI: 1.96, 4.22) and for liver-related mortality/orthotopic liver transplant (HR: 2.26; 95% CI: 1.05, 4.86) in HBV patients with features of established cirrhosis. No data on the risk of liver transplant alone were found.

#### Relationship between HBV, NAFLD, and NASH

A single article described the relationship between DM and NASH in increasing the risk of HBV disease progression.^43^ In this cross-sectional study conducted in US between 2000 and 2006, although a higher proportion of chronic HBV patients with DM than without DM had NASH (20% vs. 12%), the difference in the prevalence of NASH in chronic HBV patients with and without DM was not significant.

## Discussion

This review was based on a systematic literature search from 2000 to 2017 to identify publications about the progression of HBV-related liver disease in people with DM. Twenty articles were identified with data on HBV disease progression by DM status, most of which reported that DM was a predictor for the outcome of interest, including delayed seroclearance, cirrhosis, decompensated cirrhosis, HCC, orthotopic liver transplant/mortality and death. There were no studies about NAFLD in adults with HBV and DM, and one study showed that in people with HBV infection, the prevalence of NASH was similar regardless of DM status. The majority of the studies identified were conducted in HBV-endemic countries such as China and Taiwan, but reports from the US, Spain, Greece, France, and New Zealand were also included.

Because liver cirrhosis itself may lead to glucose intolerance and DM, the temporal relationship of DM and cirrhosis in patients with chronic HBV is unclear.^44-46^ Our searches identified 3 studies showing that DM increased the risk of cirrhosis, including 2 small case-control studies,^26,27^ and a large cross-sectional study to identify HBV infected patients with newly diagnosed DM in Taiwan.^14^ The latter study showed that the rate of cirrhosis was 1.31 per 10,000 person-years in those with DM, and 0.28 per 10,000 person-years in those without DM. Moreover, given the temporal association by which newly diagnosed DM preceded and accelerated cirrhosis and decompensated cirrhosis in chronic HBV patients, the authors suggested that a causal relationship between DM and the development of cirrhosis is possible.^14^ One study in China also showed that metabolic syndrome increased the risk of liver fibrosis in patients with chronic HBV.^29^ Although the studies indicate that DM is related to cirrhosis, in one study the association between DM and cirrhosis was not significant,^28^ suggesting that impact of DM on cirrhosis is not clear-cut.

There were 9 studies that assessed DM status and HCC with mixed results. Four studies showed that DM and/or obesity and metabolic syndrome were not associated with the development of HBV-related HCC.^12,15,31-37^ In a cohort study in Taiwan, metabolic syndrome and obesity were not significant risk factors for HCC.^31^ In a further cohort study in Taiwan, 21.6% of HBsAg-positive patients developed HCC; multivariate analysis of the entire cohort showed that HBsAg-positive serostatus and DM were independent predictors for the development of HCC, whereas among the HBsAg-positive population, DM was not an independent predictor of HCC.^34^ Finally, in studies in Korea and China, DM did not increase the incidence of HCC in patients with HBV-related cirrhosis, and in the Chinese study HBV was the only significant risk factor for HCC.^29,35^ Conversely, 4 studies reported that DM was a predictor of HCC in patients with HBV infection including a prospective study in China which showed that DM was associated with an increased risk of HCC irrespective of HBV infection status (relative risk 2.17), and among HBsAg-positive subjects, DM was the most important risk factor for HCC (relative risk 2.41).^12^ Moreover, based on the finding that the risk of HCC was 100-fold higher among chronic HBV patients with both obesity and DM than without these metabolic syndromes, the authors suggested a synergistic effect of metabolic factors and HBV infection.^12^ In a large cross-sectional study in Taiwan, the incidence of HCC in chronic HBV patients was 0.61 per 100 person-years in subjects with new-onset DM, and was 0.36 per 100 person-years in those without DM, and the multivariate analysis showed that after adjusting for factors such as age, hyperlipidemia, HBV treatment, and obesity, new onset DM was a significant predictor of HBV-related HCC (hazard ratio 1.76).^32^ Furthermore, the authors suggested a causal relationship by which DM promotes HCC in people with chronic HBV based on the finding that DM often preceded HCC by many years.^32^

There were 4 studies that reported the risk of death in HBV patients by DM status, and one study assessed the risk of orthotopic liver transplant combined with the risk of death.^37-41^ In one cohort study in the US, DM was not a predictor of hepatitis-related death in HBV patients, whereas the other studies reported that in HBV patients, DM was a predictor of all-cause death, all-cause mortality/orthotopic liver transplant and liver-related mortality/orthotopic liver transplant, as well as post-liver transplantation survival rate. More data are needed particularly regarding the impact of DM on liver transplantation outcomes in patients with HBV, although the studies suggest that DM is associated with an increased risk of death in HBV patients.

There was only one paper identified for the review which assessed the effect of DM on serological outcomes, which reported that patients with chronic HBV infection have reduced rates of HBeAg seroclearance if they have metabolic syndrome based on central obesity, dyslipidemia, hypertension, or impaired fasting glycemia.^13^ Moreover, the study reported that delayed HBeAg seroclearance was not predicted by severe hepatic steatosis, NASH, or increased BMI, suggesting that these factors may not affect seroclearance directly, although the authors could not define the pathological mechanism underlying the reduced seroclearance in metabolic syndrome.^13^

In a recent systematic review, the prevalence of NAFLD was estimated to be 25% of adults globally, 24% in North America, 23% in Europe, and 27% in Asia,^18^ yet despite this high prevalence, our review showed that there are a lack of data about progressive fatty liver disease in patients with chronic HBV infection. We identified only one study which assessed the prevalence of NASH in patients with chronic HBV with and without DM; the US-based study showed that the rate of NASH was higher in DM vs. non-DM patients and although central obesity, hypertension, and dyslipidemia were associated with NASH, there was no association between DM and NASH.^43^

This review has limitations. First, all the studies included were hospital-based, thus selecting for a sub-group of the population, and the type of DM was not always reported, although the large majority of cases were Type 2 DM. In addition, variation in the case-definitions of Type 2 DM between studies is also a confounding factor. There was also a degree of heterogeneity expected in study design, geographic location, subjects, and statistical methodology between studies. Indeed, in the studies using a retrospective study design, possible misclassification of HBV, DM status, and outcome status could have occurred. In fact, none of the studies fully described anti-HBV viral therapy or response to treatment. Finally, because of the small number of the available studies and heterogeneity of design, we did not feel that it was appropriate to provide a meta-analytic summary of different factors associated with the study outcomes.

Nevertheless, it is important to know that in most countries with a relatively low prevalence of HBV infection, HBV vaccination in adults is reimbursed on a risk-based approach with no specific guidance on adults with DM. In 2011, the US ACIP recommended HBV vaccination for adults with DM aged 19–59 years,^25^ and in 2013, the National Vaccination Committee in Belgium, recommended HBV for adults with DM aged 23–59 years.^26^ The recommendation in the US for HBV vaccination of adults with DM was based on the available clinical evidence as well as the cost-effectiveness of HBV vaccination for unvaccinated adults aged 20–59 years with DM; the cost-effectiveness model estimated that with a 10% uptake rate, 528,047 people would be vaccinated, thus preventing 4,271 HBV infections, 467 hospitalizations, 256 chronic cases, 33 cases of hepatocellular carcinoma, 13 liver transplants, and 130 deaths, with an estimated cost per QALY saved of about $75,100.^47^ Vaccination of people aged >60 years with DM was not considered to be a cost-effective strategy.^47^ However, the published cost-effectiveness model assumed that the progression of HBV disease in people with DM was the same as for non-DM individuals, whereas studies identified in this review suggest that the progression of HBV-related cirrhosis, HCC, and death may be more rapid in DM than non-DM populations. Given the profile of HBV-disease in people with DM, an updated analysis taking disease progression into account would be expected to improve the cost-effectiveness of HBV vaccination strategies.

In conclusion, our review suggests that in adults with HBV infection, DM is associated with the progression to severe liver outcomes, including cirrhosis, HCC, and death. However, most of the reviewed data on HBV assessed liver disease progression among patients who were chronically infected with HBV. Given that the probability of progression from acute to chronic HBV infection is inversely related to age, with the highest rates observed among those infected in infanthood, universal vaccination of infants is the best strategy to prevent HBV-related liver disease, HCC, and death. Nonetheless, evidence is emerging to show that adult groups, particularly subjects with DM, may benefit from HBV vaccination, although more studies are needed to better understand the relationship between metabolic syndromes and HBV-related disease to help public health authorities make informed decisions about the benefits of HBV vaccination in adults with DM and liver-diseases.

## Methods

### Searches

A systematic search of English language publications between January 1st 2000 and January 9th 2017 was performed using the PubMed, Embase, and Cochrane databases. Further searches were conducted to identify relevant gray literature including websites for World Health Organization, US Centers for Disease Prevention and Control, European Centers for Disease Prevention and Control, and The EuroHep.Net project.

There were 2 search objectives: HBV in DM subjects; and HBV in non-DM subjects. The search strings combined hepatitis B, DM, and outcome terms to retrieve articles with data on seroconversion, seroclearance, liver cirrhosis, HCC, liver transplantation, death, NASH, and NAFLD.

Studies that were relevant for the objectives were included if they had a cohort, case-control or cross-sectional design. Studies were excluded if they were non-pertinent article types (letters to the editor, editorials or comments); animal, genetic, biochemistry or molecular studies; case reports; economic evaluations; modeling studies; narrative reviews; studies with insufficient methodological quality or detail; and articles with no quantitative data. Only the most recent publications which described similar results in identical data sets were included.

### Data extraction and analyses

Relevant articles from the literature search were identified using a 3-step selection procedure. Firstly, the articles were screened by title and abstract to identify those containing relevant data, with 2 independent researchers screening 30% of the titles and abstracts in duplicate. Secondly, the full-text of the selected articles was fully assessed, to ensure compliance with the inclusion and exclusion criteria and to determine whether one of the review questions was answered. Thirdly, the articles were further screened during the data-extraction phase. If meta-analyses or good quality systematic reviews were identified, the reference lists were checked for original articles for inclusion instead of the systematic review or meta-analysis article.

The methodological quality of the articles was evaluated using the CoCanCPG checklists,^48^ which include the most important criteria on publication quality from the PRISMA and STROBE guidelines. Data were extracted into 2 tables. 1) Disease progression of hepatitis B (seroconversion, developing cirrhosis and decompensated cirrhosis, hepatocellular carcinoma, liver transplant and death) in subjects with and without DM. Articles were sorted by disease status (disease progression of subjects with hepatitis B by DM status; disease progression of subjects with DM by hepatitis B status; disease progression by hepatitis B and DM separately; and disease progression by hepatitis B only), country and first author of publication. 2) The relationship between DM and other conditions (e.g. NASH and NAFLD) in the increased risk of hepatitis B progression in subjects with DM.

The data in the studies were extracted systematically and we provide the data extraction tables and a narrative description of the results. No meta-analyses or other summary analyses were performed.

## Supplementary Material

Supplemental_Material.zip

## References

[1] World Health Organization Global Status Report on non-communicable diseases. 2014 http://www.who.int/nmh/publications/ncd-status-report-2014/en/ (accessed December 2016)

[2] World Health Organization Hepatitis B factsheet. 2015 www.who.int/mediacentre/factsheets/fs204/en/ (accessed December 2016)

[3] World Health Organization Hepatitis B vaccines: WHO position paper–recommendations. Vaccine 2010; 28(3):589-90; PMID:19896455; https://doi.org/10.1016/j.vaccine.2009.10.110 19896455

[4] Schillie SF, Xing J, Murphy TV, Hu DJ Prevalence of hepatitis B virus infection among persons with diagnosed diabetes mellitus in the United States, 1999-2010. J Viral Hepat 2012; 19(9):674-6; PMID:22863272; https://doi.org/10.1111/j.1365-2893.2012.01616.x 22863272

[5] Reilly ML, Schillie SF, Smith E, Poissant T, Vonderwahl CW, Gerard K, Baumgartner J, Mercedes L, Sweet K, Muleta D, et al. Increased risk of acute hepatitis B among adults with diagnosed diabetes mellitus. J Diabetes Sci Technol 2012; 6(4):858-66; PMID:22920812; https://doi.org/10.1177/193229681200600417 22920812 PMC3440157

[6] Collier B, Dossett LA, May AK, Diaz JJ Glucose control and the inflammatory response. Nutr Clin Pract 2008; 23(1):3-15; PMID:18203960; https://doi.org/10.1177/011542650802300103 18203960

[7] Leclercq IA, Da Silva Morais A, Schroyen B, Van Hul N, Geerts A Insulin resistance in hepatocytes and sinusoidal liver cells: Mechanisms and consequences. J Hepatol 2007; 47(1):142-56; PMID:17512085; https://doi.org/10.1016/j.jhep.2007.04.002 17512085

[8] Postic C, Dentin R, Girard J Role of the liver in the control of carbohydrate and lipid homeostasis. Diabetes Metab 2004; 30(5):398-408; PMID:15671906; https://doi.org/10.1016/S1262-3636(07)70133-7 15671906

[9] Raddatz D, Ramadori G Carbohydrate metabolism and the liver: Actual aspects from physiology and disease. Z Gastroenterol 2007; 45(1):51-62; PMID:17236121; https://doi.org/10.1055/s-2006-927394 17236121

[10] Tappy L, Minehira K New data and new concepts on the role of the liver in glucose homeostasis. Curr Opin Clin Nutr Metab Care 2001; 4(4):273-7; PMID:11458020; https://doi.org/10.1097/00075197-200107000-00005 11458020

[11] Teli T, Xanthaki D, Karalis KP Regulation of appetite and insulin signaling in inflammatory states. Ann N Y Acad Sci 2006; 1083:319-28; PMID:17148747; https://doi.org/10.1196/annals.1367.022 17148747

[12] Chen CL, Yang HI, Yang WS, Liu CJ, Chen PJ, You SL, Wang LY, Sun CA, Lu SN, Chen DS, et al. Metabolic factors and risk of hepatocellular carcinoma by chronic hepatitis B/C infection: A follow-up study in Taiwan. Gastroenterology 2008; 135(1):111-21; PMID:18505690; https://doi.org/10.1053/j.gastro.2008.03.073 18505690

[13] Hsiang JC, Wong GL, Chan HL, Chan AW, Chim AM, Wong VW Metabolic syndrome delays HBeAg seroclearance in Chinese patients with hepatitis B. Aliment Pharmacol Ther 2014; 40(6):716-26; PMID:25039861; https://doi.org/10.1111/apt.12874 25039861

[14] Huang YW, Wang TC, Lin SC, Chang HY, Chen DS, Hu JT, Yang SS, Kao JH Increased risk of cirrhosis and its decompensation in chronic hepatitis B patients with newly diagnosed diabetes: A nationwide cohort study. Clin Infect Dis 2013; 57(12):1695-702; PMID:24051864; https://doi.org/10.1093/cid/cit603 24051864

[15] Li Q, Li WW, Yang X, Fan WB, Yu JH, Xie SS, Liu L, Ma LX, Chen SJ, Kato N Type 2 diabetes and hepatocellular carcinoma: A case-control study in patients with chronic hepatitis B. Int J Cancer 2012; 131(5):1197-202; PMID:22052244; https://doi.org/10.1002/ijc.27337 22052244

[16] Bi Y, Min M, Shen W, Deng P, Du Q, Dong M, Liu Y Prognostic value of high sensitivity C-reaction protein in non-insulin dependent diabetes mellitus patients with non-alcoholic fatty liver disease. Int J Clin Exp Pathol 2015; 8(7):8494-9; PMID:26339423. 26339423 PMC4555751

[17] Shimada M, Hashimoto E, Kaneda H, Noguchi S, Hayashi N Nonalcoholic steatohepatitis: Risk factors for liver fibrosis. Hepatol Res 2002; 24(4):429-38; PMID:12479942; https://doi.org/10.1016/S1386-6346(02)00246-2 12479942

[18] Younossi ZM, Koenig AB, Abdelatif D, Fazel Y, Henry L, Wymer M Global epidemiology of nonalcoholic fatty liver disease-Meta-analytic assessment of prevalence, incidence, and outcomes. Hepatology 2016; 64(1):73-84; PMID:26707365; https://doi.org/10.1002/hep.28431 26707365

[19] Adinolfi LE, Gambardella M, Andreana A, Tripodi MF, Utili R, Ruggiero G Steatosis accelerates the progression of liver damage of chronic hepatitis C patients and correlates with specific HCV genotype and visceral obesity. Hepatology 2001; 33(6):1358-64; PMID:11391523; https://doi.org/10.1053/jhep.2001.24432 11391523

[20] Lonardo A, Adinolfi LE, Loria P, Carulli N, Ruggiero G, Day CP Steatosis and hepatitis C virus: Mechanisms and significance for hepatic and extrahepatic disease. Gastroenterology 2004; 126(2):586-97; PMID:14762795; https://doi.org/10.1053/j.gastro.2003.11.020 14762795

[21] Ong JP, Younossi ZM, Speer C, Olano A, Gramlich T, Boparai N Chronic hepatitis C and superimposed nonalcoholic fatty liver disease. Liver 2001; 21(4):266-71; PMID:11454190; https://doi.org/10.1034/j.1600-0676.2001.021004266.x 11454190

[22] Altlparmak E, Koklu S, Yalinkilic M, Yuksel O, Cicek B, Kayacetin E, Sahin T Viral and host causes of fatty liver in chronic hepatitis B. World J Gastroenterol 2005; 11(20):3056-9; PMID:15918189; https://doi.org/10.3748/wjg.v11.i20.3056 15918189 PMC4305839

[23] Cheng YL, Wang YJ, Kao WY, Chen PH, Huo TI, Huang YH, Lan KH, Su CW, Chan WL, Lin HC, et al. Inverse association between hepatitis B virus infection and fatty liver disease: a large-scale study in populations seeking for check-up. PloS One 2013; 8(8):e72049; PMID:23991037; https://doi.org/10.1371/journal.pone.0072049 23991037 PMC3750031

[24] Thomopoulos KC, Arvaniti V, Tsamantas AC, Dimitropoulou D, Gogos CA, Siagris D, Theocharis GJ, Labropoulou-Karatza C Prevalence of liver steatosis in patients with chronic hepatitis B: A study of associated factors and of relationship with fibrosis. Eur J Gastroenterol Hepatol 2006; 18(3):233-7; PMID:16462535; https://doi.org/10.1097/00042737-200603000-00002 16462535

[25] Centers for Disease Control and Prevention (CDC) Use of hepatitis B vaccination for adults with diabetes mellitus: Recommendations of the Advisory Committee on Immunization Practices (ACIP). MMWR Morb Mortal Wkly Rep 2011; 60(50):1709-11; PMID:22189894 22189894

[26] FOD Volksgezondheid en Sociale Zekerheid /SPF Santé Publique et Securité Sociale. Vaccination contre l'hépatite B 2013.

[27] Huo T, Wu JC, Hwang SJ, Lai CR, Lee PC, Tsay SH, Chang FY, Lee SD Factors predictive of liver cirrhosis in patients with chronic hepatitis B: A multivariate analysis in a longitudinal study. Eur J Gastroenterol Hepatol 2000; 12(6):687-93; PMID:10912490; https://doi.org/10.1097/00042737-200012060-00019 10912490

[28] Papatheodoridis GV, Chrysanthos N, Savvas S, Sevastianos V, Kafiri G, Petraki K, Manesis EK Diabetes mellitus in chronic hepatitis B and C: Prevalence and potential association with the extent of liver fibrosis. J Viral Hepat 2006; 13(5):303-10; PMID:16637860; https://doi.org/10.1111/j.1365-2893.2005.00677.x 16637860

[29] Wong GL, Chan HL, Yu Z, Chan AW, Choi PC, Chim AM, Chan HY, Tse CH, Wong VW Coincidental metabolic syndrome increases the risk of liver fibrosis progression in patients with chronic hepatitis B–a prospective cohort study with paired transient elastography examinations. Aliment Pharmacol Ther 2014; 39(8):883-93; PMID:24612251; https://doi.org/10.1111/apt.12658 24612251

[30] Mallet V, Hamed K, Schwarzinger M Prognosis of patients with chronic hepatitis B in France (2008-2013): A nationwide, observational and hospital-based study. J Hepatol 2017; 66(3):514-20; PMID:27826056; https://doi.org/10.1016/j.jhep.2016.10.031 27826056

[31] Chen C, Chen J, Wang J, Chang K, Tseng P, Kee K, Chen PF, Tsai LS, Chen SC, Lin SC, et al. Diabetes mellitus, metabolic syndrome and obesity are not significant risk factors for hepatocellular carcinoma in an HBV- and HCV-endemic area of Southern Taiwan. Kaohsiung J Med Sci 2013; 29:451-9; PMID:23906236; https://doi.org/10.1016/j.kjms.2012.12.006 23906236 PMC11916133

[32] Fu SC, Huang YW, Wang TC, Hu JT, Chen DS, Yang SS Increased risk of hepatocellular carcinoma in chronic hepatitis B patients with new onset diabetes: A nationwide cohort study. Aliment Pharmacol Ther 2015; 41(11):1200-9; PMID:25846548; https://doi.org/10.1111/apt.13191 25846548

[33] Ko WH, Chiu SY, Yang KC, Chen HH Diabetes, hepatitis virus infection and hepatocellular carcinoma: A case-control study in hepatitis endemic area. Hepatol Res 2012; 42(8):774-81; PMID:22469194; https://doi.org/10.1111/j.1872-034X.2012.00979.x 22469194

[34] Wang CS, Yao WJ, Chang TT, Wang ST, Chou P The impact of type 2 diabetes on the development of hepatocellular carcinoma in different viral hepatitis statuses. Cancer Epidemiol Biomarkers Prev 2009; 18(7):2054-60; PMID:19549812; https://doi.org/10.1158/1055-9965.EPI-08-1131 19549812

[35] Xiong J, Wang J, Huang J, Sun W, Wang J, Chen D Non-alcoholic steatohepatitis-related liver cirrhosis is increasing in China: A ten-year retrospective study. Clinics (Sao Paulo, Brazil) 2015; 70(8):563-8; PMID:26247669; https://doi.org/10.6061/clinics/2015(08)06 26247669 PMC4518765

[36] Seo YS, Kim MN, Kim SU, Kim SG, Um SH, Han KH, Kim YS Risk assessment of hepatocellular carcinoma using transient elastography Vs. liver biopsy in chronic hepatitis B patients receiving antiviral therapy. Medicine 2016; 95(12):e2985; PMID:27015173; https://doi.org/10.1097/MD.0000000000002985 27015173 PMC4998368

[37] Hsiang JC, Gane EJ, Bai WW, Gerred SJ Type 2 diabetes: A risk factor for liver mortality and complications in hepatitis B cirrhosis patients. J Gastroenterol Hepatol 2015; 30(3):591-9; PMID:25250942; https://doi.org/10.1111/jgh.12790 25250942

[38] Huo TI, Wu JC, Lui WY, Lee PC, Huang YH, Chau GY, Tsay SH, Chang FY, Lee SD Diabetes mellitus is a recurrence-independent risk factor in patients with hepatitis B virus-related hepatocellular carcinoma undergoing resection. Eur J Gastroenterol Hepatol 2003; 15(11):1203-8; PMID:14560154; https://doi.org/10.1097/00042737-200311000-00009 14560154

[39] Ribes J, Cleries R, Rubio A, Hernandez JM, Mazzara R, Madoz P, Casanovas T, Casanova A, Gallen M, Rodríguez C, et al. Cofactors associated with liver disease mortality in an HBsAg-positive Mediterranean cohort: 20 years of follow-up. Int J Cancer 2006; 119(3):687-94; PMID:16496403; https://doi.org/10.1002/ijc.21882 16496403

[40] Stepanova M, Rafiq N, Younossi ZM Components of metabolic syndrome are independent predictors of mortality in patients with chronic liver disease: A population-based study. Gut 2010; 59(10):1410-5; PMID:20660697; https://doi.org/10.1136/gut.2010.213553 20660697

[41] Szpakowski JL, Tucker LY Causes of death in patients with hepatitis B: A natural history cohort study in the United States. Hepatology (Baltimore, Md) 2013; 58(1):21-30; PMID:23080403; https://doi.org/10.1002/hep.26110 23080403

[42] Ling Q, Xu X, Wei Q, Wei X, Wang Z, Zhou L, Zheng S Impact of preexisting diabetes mellitus on outcome after liver transplantation in patients with hepatitis B virus-related liver disease. Dig Dis Sci 2011; 56(3):889-93; PMID:20703811; https://doi.org/10.1007/s10620-010-1358-3 20703811

[43] Bondini S, Kallman J, Wheeler A, Prakash S, Gramlich T, Jondle DM, Younossi ZM Impact of non-alcoholic fatty liver disease on chronic hepatitis B. Liver Int 2007; 27(5):607-11; PMID:17498244; https://doi.org/10.1111/j.1478-3231.2007.01482.x 17498244

[44] Hsieh PS, Hsieh YJ Impact of liver diseases on the development of type 2 diabetes mellitus. World J Gastroenterol 2011; 17(48):5240-5; PMID:22219592; https://doi.org/10.3748/wjg.v17.i48.5240 22219592 PMC3247687

[45] Kingston ME, Ali MA, Atiyeh M, Donnelly RJ Diabetes mellitus in chronic active hepatitis and cirrhosis. Gastroenterology 1984; 87(3):688-94; PMID:6086443. 6086443

[46] Megyesi C, Samols E, Marks V Glucose tolerance and diabetes in chronic liver disease. Lancet (London, England) 1967; 2(7525):1051-6; PMID:4168535; https://doi.org/10.1016/S0140-6736(67)90334-0 4168535

[47] Hoerger TJ, Schillie S, Wittenborn JS, Bradley CL, Zhou F, Byrd K, Murphy TV Cost-effectiveness of hepatitis B vaccination in adults with diagnosed diabetes. Diabetes Care 2013; 36(1):63-9; PMID:22933435; https://doi.org/10.2337/dc12-0759 22933435 PMC3526214

[48] Coordination of Cancer Clinical Practice Guidelines in Europe www.cocancpg.eu/ (accessed December 2016)

