# Supplementary material for: Should adults with diabetes mellitus be vaccinated against hepatitis B virus? A systematic review of diabetes mellitus and the progression of hepatitis B disease
Source: Hum Vaccin Immunother. 2017 Jul 25;13(11):2695–706. doi: 10.1080/21645515.2017.1353850 (PMC5703367; doi:10.1080/21645515.2017.1353850)
Supplement: Supplemental_Material.zip [file khvi-13-11-1353850-s001.zip › Younossi et al. - supplement 2.docx]

Supplement 2. Search string

1. **Pubmed**
2. *Disease progression of hepatitis B in subjects with DM*

"Hepatitis B"[Mesh] OR “Hepatitis B”[tiab] OR HBV[tiab] OR Hep B[tiab] AND "Diabetes Mellitus"[Mesh] OR diabe*[tiab] OR DM[tiab] AND "Liver Cirrhosis"[Mesh] OR cirrhotic*[tiab] OR cirrhosis[tiab] OR cirrhoses[tiab] OR "Carcinoma, Hepatocellular"[Mesh] OR “Hepatocellular Carcinoma”[tiab] OR “Hepatocellular Carcinomas”[tiab] OR “Liver Cell Carcinoma”[tiab] OR “Liver Cell Carcinomas”[tiab] OR Hepatoma[tiab] OR Hepatomas [tiab] OR hepatocarcinoma*[tiab] OR HCC[tiab] OR "Liver Transplantation"[Mesh] OR ((Liver[tiab] OR livers[tiab] OR hepatic*[tiab]) AND (Transplant*[tiab] OR grafting*[tiab])) OR "Death"[Mesh] OR death[tiab] OR dying[tiab] OR died[tiab] OR die[tiab] OR mortal*[tiab] OR nonalcoholic fatty liver disease[Mesh] OR nonalcoholic steatohepatitis[tiab] OR NASH[tiab] OR nonalcoholic fatty liver disease[tiab] OR NAFLD[tiab] OR seroconversion[tiab] OR seroclearance[tiab]

Limit: Publication date from 1^st^ January 2000 to 2^nd^ December 2015

1. *Disease progression of hepatitis B in subjects without DM*

"Hepatitis B"[Mesh] OR “Hepatitis B”[tiab] OR HBV[tiab] OR Hep B[tiab] AND Health*[tiab] AND "Liver Cirrhosis"[Mesh] OR cirrhotic*[tiab] OR cirrhosis[tiab] OR cirrhoses[tiab] OR "Carcinoma, Hepatocellular"[Mesh] OR “Hepatocellular Carcinoma”[tiab] OR “Hepatocellular Carcinomas”[tiab] OR “Liver Cell Carcinoma”[tiab] OR “Liver Cell Carcinomas”[tiab] OR Hepatoma[tiab] OR Hepatomas [tiab] OR hepatocarcinoma*[tiab] OR HCC[tiab] OR "Liver Transplantation"[Mesh] OR ((Liver[tiab] OR livers[tiab] OR hepatic*[tiab]) AND (Transplant*[tiab] OR grafting*[tiab])) OR "Death"[Mesh] OR death[tiab] OR dying[tiab] OR died[tiab] OR die[tiab] OR mortal*[tiab] OR nonalcoholic fatty liver disease[mesh] OR nonalcoholic steatohepatitis[tiab] OR NASH[tiab] OR nonalcoholic fatty liver disease[tiab] OR NAFLD[tiab] OR seroconversion[tiab] OR seroclearance[tiab]

Limit: Publication date from 1^st^ January 2000 to 2^nd^ December 2015

*c) Relationship between HepB and NAFLD/NASH*

"Hepatitis B"[Mesh] OR “Hepatitis B”[tiab] OR HBV[tiab] OR Hep B[tiab] AND Nonalcoholic fatty liver disease[Mesh] OR nonalcoholic steatohepatitis[tiab] OR NASH[tiab] OR nonalcoholic fatty liver disease[tiab] OR NAFLD[tiab]

Limit: Publication date from 1^st^ January 2000 to 3^rd^ December 2015

### Embase

1. *Disease progression of hepatitis B in subjects with DM*

'hepatitis B'/exp OR ‘Hepatitis B’:ti,ab OR HBV:ti,ab OR ‘Hep B’:ti,ab AND 'diabetes mellitus'/exp OR diabe*:ti,ab OR DM:ti,ab AND 'liver cirrhosis'/exp OR cirrhotic*:ti,ab OR cirrhosis:ti,ab OR cirrhoses:ti,ab OR 'liver cell carcinoma'/exp OR ‘hepatocellular carcinoma’:ti,ab OR ‘hepatocellular carcinomas’:ti,ab OR ‘liver cell carcinoma’:ti,ab OR ‘liver cell carcinomas’:ti,ab OR hepatoma:ti,ab OR hepatomas:ti,ab OR hepatocarcinoma*:ti,ab OR HCC:ti,ab OR 'liver transplantation'/exp OR ((liver:ti,ab OR livers:ti,ab OR hepatic*:ti,ab) AND (transplant*:ti,ab OR grafting*:ti,ab)) OR 'death'/exp OR death:ti,ab OR dying:ti,ab OR died:ti,ab OR die:ti,ab OR mortal*:ti,ab OR 'nonalcoholic fatty liver'/exp OR ‘nonalcoholic fatty liver disease’:ti,ab OR NAFLD:ti,ab OR ‘nonalcoholic steohepatitis’:ti,ab OR NASH:ti,ab OR 'seroconversion'/exp OR seroconversion:ti,ab OR seroclearance:ti,ab

Limit: Publication date from 1^st^ January 2000 to 2^nd^ December 2015

1. *Disease progression of hepatitis B in subjects without DM*

'hepatitis B'/exp OR ‘Hepatitis B’:ti,ab OR HBV:ti,ab OR ‘Hep B’:ti,ab AND Health*:ti,ab AND 'liver cirrhosis'/exp OR cirrhotic*:ti,ab OR cirrhosis:ti,ab OR cirrhoses:ti,ab OR 'liver cell carcinoma'/exp OR ‘hepatocellular carcinoma’:ti,ab OR ‘hepatocellular carcinomas’:ti,ab OR ‘liver cell carcinoma’:ti,ab OR ‘liver cell carcinomas’:ti,ab OR hepatoma:ti,ab OR hepatomas:ti,ab OR hepatocarcinoma*:ti,ab OR HCC:ti,ab OR 'liver transplantation'/exp OR ((liver:ti,ab OR livers:ti,ab OR hepatic*:ti,ab) AND (transplant*:ti,ab OR grafting*:ti,ab)) OR 'death'/exp OR death:ti,ab OR dying:ti,ab OR died:ti,ab OR die:ti,ab OR mortal*:ti,ab OR 'nonalcoholic fatty liver'/exp OR ‘nonalcoholic fatty liver disease’:ti,ab OR NAFLD:ti,ab OR ‘nonalcoholic steohepatitis’:ti,ab OR NASH:ti,ab OR 'seroconversion'/exp OR seroconversion:ti,ab OR seroclearance:ti,ab

Limit: Publication date from 1^st^ January 2000 to 2^nd^ December 2015

*c) Relationship between HepB and NAFLD/NASH*

'hepatitis B'/exp OR ‘Hepatitis B’:ti,ab OR HBV:ti,ab OR ‘Hep B’:ti,ab AND *Outcomes* 'nonalcoholic fatty liver'/exp OR ‘nonalcoholic fatty liver disease’:ti,ab OR NAFLD:ti,ab OR ‘nonalcoholic steohepatitis’:ti,ab OR NASH:ti,ab

Limit: Publication date from 1^st^ January 2000 to 3^rd^ December 2015

### Cochrane library

The search was performed on 12^th^ February 2015 and 3^rd^ December 2015.

1. *Disease progression of hepatitis B in subjects with DM*

("hepatitis B":ti or "hepatitis B":ab or HBV:ti or HBV:ab or "hep B":ti or "hep B":ab or "serum hepatitis":ti or "serum hepatitis":ab) OR MeSH descriptor: [hepatitis B] explode all trees OR MeSH descriptor: [hepatitis B virus] explode all trees AND (DM:ti or DM:ab or diabe*:ti or diabe*:ab or IDDM:ti or IDDM:ab or NIDDM:ti or NIDDM:ab)OR MeSH descriptor: [diabetes mellitus] explode all trees AND (cirrhotic*:ti or cirrhotic*:ab or cirrhosis:ti or cirrhosis:ab or cirrhoses:ti or cirrhoses:ab) OR MeSH descriptor: [liver cirrhosis] explode all trees OR ("hepatocellular carcinoma":ti or "hepatocellular carcinoma":ab or "hepatocellular carcinomas":ti or "hepatocellular carcinomas":ab or "liver cell carcinoma":ti or "liver cell carcinoma":ab or hepatoma:ti or hepatoma:ab or hepatomas:ti or hepatomas:ab or hepatocarcinoma*:ti or hepatocarcinoma*:ab or HCC:ti or HCC:ab) OR MeSH descriptor: [carcinoma, hepatocellular] explode all trees ((liver:ti or liver:ab or livers:ti or livers:ab or hepatic:ti or hepatic:ab) and (transplant*:ti or transplant*:ab or grafting*:ti or grafting*:ab)) OR MeSH descriptor: [liver transplantation] explode all trees OR (death:ti or death:ab or dying:ti or dying:ab or died:ti or died:ab or die:ti or die:ab or mortal*:ti or mortal*:ab) OR MeSH descriptor: [death] explode all trees OR ("Non-alcoholic Fatty Liver Disease":ti or "Non-alcoholic Fatty Liver Disease":ab or "nonalcoholic steatohepatitis":ti or "nonalcoholic steatohepatitis":ab or NASH:ti or NASH:ab or NAFLD:ti or NAFLD:ab) OR MeSH descriptor: [Non-alcoholic Fatty Liver Disease] explode all trees OR (seroconversion:ti or seroconversion:ab)OR (seroclearance:ti or seroclearance:ab)

1. *Disease progression of hepatitis B in subjects without DM*

("hepatitis B":ti or "hepatitis B":ab or HBV:ti or HBV:ab or "hep B":ti or "hep B":ab or "serum hepatitis":ti or "serum hepatitis":ab) OR MeSH descriptor: [hepatitis B] explode all trees OR MeSH descriptor: [hepatitis B virus] explode all trees AND **(**health*:ti or health*:ab) AND (cirrhotic*:ti or cirrhotic*:ab or cirrhosis:ti or cirrhosis:ab or cirrhoses:ti or cirrhoses:ab) OR MeSH descriptor: [liver cirrhosis] explode all trees ("hepatocellular carcinoma":ti or "hepatocellular carcinoma":ab or "hepatocellular carcinomas":ti or "hepatocellular carcinomas":ab or "liver cell carcinoma":ti or "liver cell carcinoma":ab or hepatoma:ti or hepatoma:ab or hepatomas:ti or hepatomas:ab or hepatocarcinoma*:ti or hepatocarcinoma*:ab or HCC:ti or HCC:ab or seroconversion:ti or seroconversion:ab or seroclearance:ti or seroclearance:ab) OR MeSH descriptor: [carcinoma, hepatocellular] explode all trees OR ((liver:ti or liver:ab or livers:ti or livers:ab or hepatic:ti or hepatic:ab) and (transplant*:ti or transplant*:ab or grafting*:ti or grafting*:ab)) OR MeSH descriptor: [liver transplantation] explode all trees OR (death:ti or death:ab or dying:ti or dying:ab or died:ti or died:ab or die:ti or die:ab or mortal*:ti or mortal*:ab) OR MeSH descriptor: [death] explode all trees OR ("Non-alcoholic Fatty Liver Disease":ti or "Non-alcoholic Fatty Liver Disease":ab or "nonalcoholic steatohepatitis":ti or "nonalcoholic steatohepatitis":ab or NASH:ti or NASH:ab or NAFLD:ti or NAFLD:ab) OR MeSH descriptor: [Non-alcoholic Fatty Liver Disease] explode all trees OR (seroconversion:ti or seroconversion:ab) OR (seroclearance:ti or seroclearance:ab)

*c) Relationship between HepB and NAFLD/NASH*

("hepatitis B":ti or "hepatitis B":ab or HBV:ti or HBV:ab or "hep B":ti or "hep B":ab or "serum hepatitis":ti or "serum hepatitis":ab) OR MeSH descriptor: [hepatitis B] explode all trees OR MeSH descriptor: [hepatitis B virus] explode all trees AND ("Non-alcoholic Fatty Liver Disease":ti or "Non-alcoholic Fatty Liver Disease":ab or "nonalcoholic steatohepatitis":ti or "nonalcoholic steatohepatitis":ab or NASH:ti or NASH:ab or NAFLD:ti or NAFLD:ab) OR MeSH descriptor: [Non-alcoholic Fatty Liver Disease] explode all trees
