# Supplementary material for: Should adults with diabetes mellitus be vaccinated against hepatitis B virus? A systematic review of diabetes mellitus and the progression of hepatitis B disease
Source: Hum Vaccin Immunother. 2017 Jul 25;13(11):2695–706. doi: 10.1080/21645515.2017.1353850 (PMC5703367; doi:10.1080/21645515.2017.1353850)
Supplement: Supplemental_Material.zip [file khvi-13-11-1353850-s001.zip › Younossi et al. - supplement 1.docx]

**Supplement 1. Results from articles included**

| **Reference** | **Subjects (N)** | **Age (years)** | **Gender** | **Results by outcome** | **Authors’ conclusions** |
| --- | --- | --- | --- | --- | --- |
| Hsiang  et al. 2014^1^ | 413 | Normal (n=158): 35 ± 10  Pre-MetS (n=194): 42 ± 12  MetS: (n=61): 49 ± 10 | Males  Normal: 56.3%  Pre-MetS: 60.3% MetS: 75% | **HBeAg seroclearance:**  DM at baseline: HR = 0.55 (95% CI: 0.32–0.97) | Baseline DM: predictor for delayed HBeAg seroclearance |
| Huang et al. 2013^2^ | DM: 351  Non-DM:7,886 | DM: 49.04 ± 11.55  Non-DM: 37.04 ± 11.96 | Males  DM : 59.5%  Non-DM: 60.9% | **Cirrhosis & decompensated cirrhosis:** Newly diagnosed DM as an independent predictor of cirrhosis (HR: 2.015; 95% CI: 1.393–2.915) and decompensated cirrhosis (HR: 1.792; 95% CI, 1.192–2.695) in CHB patientswith newly diagnosed DM.  Proportion of CHB patients with cirrhosis after follow-up:  DM cohort: 10.8% (38/351)  Non-DM cohort: 2.6% (208/7,883)  Incidence rate of cirrhosis during follow-up:  DM cohort: 1.31 per 10,000 person-years  Non-DM cohort: 0.28 per 10,000 person-years | Newly diagnosed diabetes is an independent predictor for cirrhosis development and its decompensation in CHB patients |
| Huo et al. 2000^3^ | 516 | 42 ± 15 | 80.0% males | **Cirrhosis:** DM to be associated with cirrhosis development (OR: 5.2; 95% CI: 2.0–13.5).  Incidence of cirrhosis in chronic HBV patients:  Incidence: 13.8% (71/516)  Calculated annual incidence: 2.4% | HBV-infected patients with diabetes have increased risk of liver cirrhosis. |
| Papatheodoridis et al, 2006^4^ | 174  DM: 25  non-DM :149 | DM: 59.4 ± 10.0  Non-DM: 50.3 ± 13.4 | Males  DM: 84.0%  Non-DM: 73.8% | **Cirrhosis:** DM to be associated with cirrhosis in HBeAg negative CHB patients (model 1OR: 2.96; 95% CI: 0.95–9.22; model 2OR: 3.87; 95% CI: 1.31–11.45).  Proportion of DM in CHB patients: 14% (25/174) | The presence of diabetes is strongly associated with more severe liver fibrosis, but such an association may be related to the high prevalence of diabetes in patients with cirrhosis. |
| Wong et al. 2014^5^ | 663 (CHB-DM n=70; CHB-non DM n=593) | 43 ± 12 | 55% males | **Cirrhosis:** The OR for liver fibrosis progression in those with DM at 44 months follow-up versus patients with resolved DM was 1.1 (95% CI: 0.5, 2.2: p=0.87). | No conclusion regarding liver fibrosis progression by DM status as the main objective of the study was on metabolic syndrome. |
| Chen et al. 2013^6^ | 56, 231 (5,606 HBV) | <65: 59 %  >65: 41% | 30.9 % males | **HCC:** DM was not a significant predictor for HCC in hepatitis B subjects (HR: 1.61; 95% CI: 0.73–3.58).  Risk factors for incident HCC using the Cox proportional Hazard model:  HBV: 21.8% (57/262)  Non-DM: 87% (228/262)  DM: 13% (34/262)  Risk factors for incident HCC in the HBV carrier DM group: 12.3 % (7/57) | HepB was significantly associated with the occurrence of HCC; DM was not significantly associated with the occurrence of HCC.  DM was not a significant risk factor for HCC, regardless of HepB status |
| Wang et al. 2009^7^ | 5,929 (696 HBsAg positive) | 49.3 ± 11 | 51.3 % males | **HCC:** DM was not a significant predictor for HCC in hepatitis B subjects (HR: 1.3; 95% CI: 0.3–5.6).  Prevalence of HBsAg positive:  11.7% (696/5,929)  Mean prevalence of DM before study among HBsAg positive participants:  6.8% (47/696)  Proportion of individuals who developed HCC during the follow-up among HBsAg positive participants:  21.6% (24/696) | A multivariate Cox proportional hazards model analysis showed that HBsAg positive and DM were independent predictors for the development of HCC.  A multivariate Cox proportional hazard model analysis stratified by HBsAg positive showed that DM was not an independent predictor for HCC in HBsAg positive participants. |
| Chen et al. 2008^8^ | 3, 931 | 45.9 ± 9.8 | 50.9% males | **HCC:** DM was associated with HCC in HBsAg positive subjects (RR: 2.41; 95%CI: 1.17–4.95). Incidence of HCC per 10^5^ person-years among chronic HBV participants:  387.3 | For HBsAg positive participants, diabetes was associated with a HCC RR of 2.17. In the multivariate-adjusted analysis, for HBsAg-seropositive subjects, history of diabetes was the most important HCC risk factor. |
| Li et al. 2012^9^ | Cases: 1,105  Controls: 5,170 | Cases: 53.8 ± 9.3 Controls: 44.9 ± 10.7 | Males  Cases: 84.7%  Controls: 73.8% | **HCC:** DM was significantly associated with HCC in female CHB patients (OR: 1.9; 95% CI: 1.1–3.4). The prevalence of DM was higher among HCC patients without cirrhosis than among those with cirrhosis (12.1% vs. 6.7%). Restricted analyses among female patients without cirrhosis indicated that DM was strongly associated with HCC risk with adjusted OR of 5.6 (2.2–14.1). | Patients with CHB and DM were a significant risk factor for HCC particularly in women (seen with caution, due to the small sample size of women). |
| Ko et al. 2012^10^ | Cases: 369  Controls:1,536 | Cases: 62.6  Controls: 48.2 | Males  Cases: 73.4%  Controls: 55.4% | **HCC:** Joint effect of HBV and diabetes on the risk of HCC (OR: 4.32; 95% CI: 1.92–9.70).  The association between DM and the risk for HCC was significant regardless of the presence of HBV infection. In the absence of both hepatitis virus infections, the independent effect of DM accounted for 7.5% risk for HCC from the underlying population. | The effect of diabetes on the risk for developing HCC is synergistic with HBV |
| Fu et al. 2015^11^ | 4,179  (CHB-DM cohort: 2,099; CHB non-DM cohort: 2,080) | CHB-DM cohort: 49.19 ± 12.48  CHB non-DM cohort: 49.05 ± 12.42 | Males  CHB-DM cohort: 58.55%  CHB non-DM cohort: 58.37% | **HCC:** Risk of HCC in CHB patients with new onset DM (HR: 1.798; 95% CI: 1.194–2.707).  A higher proportion of patients in CHB-DM cohort developed HCC as compared to those in CHB non-DM (3.29% vs. 2.02%). More patients in CHB-DM cohort had cirrhosis (16.29% vs. 12.88%) | DM accelerates HCC development in CHB patients. CHB patients who develop DM during follow-up are at an increased risk of HCC over time. |
| Xiong et al. 2015^12^ | 1,582  (HBV-related Liver Cirrhosis patients: 938) | 52.8 ± 12.8 | 69.4% males | **HCC:** In cirrhosis patients with HBV and DM increased trend of HCC was observed (OR: 1.27; 95% CI: 0.53–3.07).  Risk factors for HCC  among patients with cirrhosis induced only by HBV (n=938), a trend of increased rates of HCC in DM patients was found (7.1% vs. 6.7%) | When considering only the HBV-related cirrhotic subgroup, a statistically insignificant trend of increased HCC in patients with DM was seen. |
| Seo et al. 2016^13^ | 381 (CHB-DM n=31; CHB-non-DM n=350) | 44.1 | 65.9% males | **HCC:** HCC was higher in those with DM than those without (20.6% versus 6.9%, respectively), and univariate analysis showed that DM was a significant predictor for HCC (p=0.015) | No conclusion specific for the results relevant for the review objectives |
| Mallet et al. 2017^14^ | 48,189 CHB | 44 (range, 32–57) | 58.6% males | **HCC.** The multivariate analysis showed that DM was a risk factor for liver disease progression with an adjusted HR of 1.40 (95% CI: 1.32, 1.48) | No conclusion regarding liver fibrosis progression by DM status as the main objective of the study was assessed risk factors for a composite outcome of end-stage liver disease and/or HCC |
| Stepanova et al. 2010^15^ | 66 | 43.1 ± 2.9 | 65.2 ± 8.8% males | **Mortality:** Diabetes as independent predictor of overall mortality in the CHB cohort:  Adjusted HR: 30.79 (8.36–113.42) | DM is an independent risk factor of overall mortality in CHB patients. |
| Hsiang et al. 2015^16^ | 223 | 51 ± 12 | 66.8% males | **Liver transplant/Mortality:**  DM was a predictor of liver mortality or OLT (HR: 2.26; CI: 1.05–4.86), and overall mortality or OLT (HR: 2.25;  CI: 1.96–4.22).  Proportion of DM at baseline:  DM: 22.4% (50/223)  Non-DM: 77.6 % (173/223) | DM was an independent predictor of HCC development, liver-related mortality/orthotopic liver transplant, and all- cause mortality in patients with HepB cirrhosis.  DM is a poor prognostic factor on cirrhosis complications and mortality in a cohort of HBV cirrhosis patients. |
| Szpakowski et al. 2013^17^ | 6,689 | 41.3 ± 13.8 | NR | **Mortality:** DM as a predictor of all-cause mortality (HR: 1.5; 95% CI: 1.2–1.8) and HBV-related death (HR: 1.3; 95% CI: 0.9–1.8). | Diabetes is a predictor of all-cause mortality, but not of HBV-related death. |
| Huo et al. 2003^18^ | 239 | 61 ± 13 (range 20-85) | 87.9% males | **Mortality:** RR for mortality in HCC patients according to status of HBsAg and diabetes:  DM, HBsAg positive RR: 2.1 (1.1-4.6)  DM, HBsAg negative RR: 1.2 (0.4-3.9) | DM patients with HBV-related HCC may have a poor prognosis.  DM HCC patients seropositive for HBsAg had the highest risk of mortality compared with other groups of patients. |
| Ribes et al. 2006^19^ | Cases: 51  Controls: 512 | 33.1 ± 10.7 | 100% males | **Mortality:** Age-adjusted ORs for the risk of death from liver disease in HBsAg positive men (OR: 3.6; 95% CI:1.3–9.6) | Diabetes increases the risk of death from liver disease in HBsAg positive men. |
| Ling et al. 2011^20^ | Cases: 48  Controls: 96 | Cases: 45.1 ± 7.5  Controls: 45.5 ± 7.2 | Males  Cases: 89.6%  Controls: 89.6% | **Mortality:** Patient cumulative survivals were not significantly different between the DM and control group | Pre-existing DM is not a contraindication for liver transplantation in patients with HBV-related liver disease. |
| Bondini et al. 2007^21^ | 153 | 50.5 ± 27.5 | 66% males | **NASH:** | No conclusion specific for the results relevant for the review objectives. |

CHB: chronic hepatitis B; CI: confidence interval; DM: diabetes mellitus; HBV: hepatitis B virus; HBeAg: Hepatitis B e antigen; HBsAg: hepatitis B surface antigen; HCC: hepatocellular carcinoma; HepB: hepatitis B; HR: hazard ratio; MetS: Metabolic Syndrome; NASH: non-alcoholic steatohepatitis; OLT: orthopic liver transplant; OR: odds ratio; RR: relative risk

1. Hsiang JC, Wong GL, Chan HL, Chan AW, Chim AM, Wong VW. Metabolic syndrome delays HBeAg seroclearance in Chinese patients with hepatitis B. Alimentary pharmacology & therapeutics. 2014;40(6):716-26.

2. Huang YW, Wang TC, Lin SC, Chang HY, Chen DS, Hu JT, et al. Increased risk of cirrhosis and its decompensation in chronic hepatitis B patients with newly diagnosed diabetes: a nationwide cohort study. Clinical infectious diseases : an official publication of the Infectious Diseases Society of America. 2013;57(12):1695-702.

3. Huo T, Wu JC, Hwang SJ, Lai CR, Lee PC, Tsay SH, et al. Factors predictive of liver cirrhosis in patients with chronic hepatitis B: a multivariate analysis in a longitudinal study. European journal of gastroenterology & hepatology. 2000;12(6):687-93.

4. Papatheodoridis GV, Chrysanthos N, Savvas S, Sevastianos V, Kafiri G, Petraki K, et al. Diabetes mellitus in chronic hepatitis B and C: prevalence and potential association with the extent of liver fibrosis. Journal of viral hepatitis. 2006;13(5):303-10.

5. Wong GL, Chan HL, Yu Z, Chan AW, Choi PC, Chim AM, et al. Coincidental metabolic syndrome increases the risk of liver fibrosis progression in patients with chronic hepatitis B--a prospective cohort study with paired transient elastography examinations. Alimentary pharmacology & therapeutics. 2014;39(8):883-93.

6. Chen C, Chen J, Wang J, Chang K, Tseng P, Kee K, et al. Diabetes mellitus, metabolic syndrome and obesity are not significant risk factors for hepatocellular carcinoma in an HBV- and HCV-endemic area of Southern Taiwan. Kaohsiung J Med Sci. 2013;29(451-459).

7. Wang CS, Yao WJ, Chang TT, Wang ST, Chou P. The impact of type 2 diabetes on the development of hepatocellular carcinoma in different viral hepatitis statuses. Cancer epidemiology, biomarkers & prevention : a publication of the American Association for Cancer Research, cosponsored by the American Society of Preventive Oncology. 2009;18(7):2054-60.

8. Chen CL, Yang HI, Yang WS, Liu CJ, Chen PJ, You SL, et al. Metabolic factors and risk of hepatocellular carcinoma by chronic hepatitis B/C infection: a follow-up study in Taiwan. Gastroenterology. 2008;135(1):111-21.

9. Li Q, Li WW, Yang X, Fan WB, Yu JH, Xie SS, et al. Type 2 diabetes and hepatocellular carcinoma: a case-control study in patients with chronic hepatitis B. International journal of cancer. 2012;131(5):1197-202.

10. Ko WH, Chiu SY, Yang KC, Chen HH. Diabetes, hepatitis virus infection and hepatocellular carcinoma: A case-control study in hepatitis endemic area. Hepatology research : the official journal of the Japan Society of Hepatology. 2012;42(8):774-81.

11. Fu SC, Huang YW, Wang TC, Hu JT, Chen DS, Yang SS. Increased risk of hepatocellular carcinoma in chronic hepatitis B patients with new onset diabetes: a nationwide cohort study. Alimentary pharmacology & therapeutics. 2015;41(11):1200-9.

12. Xiong J, Wang J, Huang J, Sun W, Wang J, Chen D. Non-alcoholic steatohepatitis-related liver cirrhosis is increasing in China: a ten-year retrospective study. Clinics (Sao Paulo, Brazil). 2015;70(8):563-8.

13. Seo YS, Kim MN, Kim SU, Kim SG, Um SH, Han KH, et al. Risk Assessment of Hepatocellular Carcinoma Using Transient Elastography Vs. Liver Biopsy in Chronic Hepatitis B Patients Receiving Antiviral Therapy. Medicine. 2016;95(12):e2985.

14. Mallet V, Hamed K, Schwarzinger M. Prognosis of patients with chronic hepatitis B in France (2008-2013): A nationwide, observational and hospital-based study. Journal of hepatology. 2017;66(3):514-20.

15. Stepanova M, Rafiq N, Younossi ZM. Components of metabolic syndrome are independent predictors of mortality in patients with chronic liver disease: a population-based study. Gut. 2010;59(10):1410-5.

16. Hsiang JC, Gane EJ, Bai WW, Gerred SJ. Type 2 diabetes: a risk factor for liver mortality and complications in hepatitis B cirrhosis patients. Journal of gastroenterology and hepatology. 2015;30(3):591-9.

17. Szpakowski JL, Tucker LY. Causes of death in patients with hepatitis B: a natural history cohort study in the United States. Hepatology (Baltimore, Md). 2013;58(1):21-30.

18. Huo TI, Wu JC, Lui WY, Lee PC, Huang YH, Chau GY, et al. Diabetes mellitus is a recurrence-independent risk factor in patients with hepatitis B virus-related hepatocellular carcinoma undergoing resection. European journal of gastroenterology & hepatology. 2003;15(11):1203-8.

19. Ribes J, Cleries R, Rubio A, Hernandez JM, Mazzara R, Madoz P, et al. Cofactors associated with liver disease mortality in an HBsAg-positive Mediterranean cohort: 20 years of follow-up. International journal of cancer. 2006;119(3):687-94.

20. Ling Q, Xu X, Wei Q, Wei X, Wang Z, Zhou L, et al. Impact of preexisting diabetes mellitus on outcome after liver transplantation in patients with hepatitis B virus-related liver disease. Digestive diseases and sciences. 2011;56(3):889-93.

21. Bondini S, Kallman J, Wheeler A, Prakash S, Gramlich T, Jondle DM, et al. Impact of non-alcoholic fatty liver disease on chronic hepatitis B. Liver international : official journal of the International Association for the Study of the Liver. 2007;27(5):607-11.
